# Coal to Clean: Comparing Advanced Electrodes for Desulfurization and Copper Recovery

**DOI:** 10.3390/ma17194790

**Published:** 2024-09-29

**Authors:** Katarina R. Pantović Spajić, Marijana R. Pantović Pavlović, Srecko Stopic, Vesna S. Cvetković, Nataša M. Petrović, Branislav Marković, Miroslav M. Pavlović

**Affiliations:** 1 Institute for Technology of Nuclear and Other Mineral Raw Materials, 11000 Belgrade, Serbia; k.pantovic@itnms.ac.rs (K.R.P.S.); b.markovic@itnms.ac.rs (B.M.); 2 Department of Electrochemistry, Institute of Chemistry, Technology and Metallurgy, National Institute of Republic of Serbia, University of Belgrade, 11000 Belgrade, Serbia; v.cvetkovic@ihtm.bg.ac.rs (V.S.C.); vukicevic@ihtm.bg.ac.rs (N.M.P.); miroslav.pavlovic@ihtm.bg.ac.rs (M.M.P.); 3 IME Process Metallurgy and Metal Recycling, RWTH Aachen University, 52072 Aachen, Germany

**Keywords:** electrochemical desulfurization, dimensionally stable anode (DSA), graphite electrode, sulfur removal, copper recovery, energy efficiency, coal treatment

## Abstract

This study explores the electrochemical desulfurization of coal and the recovery of copper (Cu) using dimensionally stable anode (DSA) electrodes. Background: The research addresses the need for effective sulfur removal from coal to reduce emissions. Methods: Electrochemical desulfurization was conducted using DSA and graphite electrodes, evaluating parameters like activation energy, desulfurization rate, and energy consumption. Cyclic voltammetry and linear sweep voltammetry were used to study the electrochemical properties. Results: The DSA electrode demonstrated superior performance with higher desulfurization rates, lower activation energy, and better response to temperature increases compared to the graphite electrode. Optimal desulfurization was achieved at 50 °C with the DSA electrode, balancing efficiency and energy consumption. Copper recovery from the solution post-desulfurization was effective, with an 86.34% recovery rate at −0.15 V vs. (Ag|AgCl). The energy consumption for the Cu recovery was calculated to be 10.56 J, and the total cost for recovering 1 ton of Cu was approximately 781.20 €. Conclusions: The study highlights the advantages of DSA electrodes for efficient sulfur removal and metal recovery, promoting cleaner energy production and environmental sustainability. Future research should focus on optimizing electrochemical conditions and scaling up the process for industrial applications.

## 1. Introduction

The presence of elevated sulfur levels in coal introduces detrimental consequences [1,2]. The burgeoning demand for low-sulfur coal as a viable alternative fuel was compounded by the recent COVID-19 pandemic, leading to a surplus in high-sulfur content coal supply. This incongruity between the overall supply of low-sulfur coal and escalating demand underscores the imperative for high-sulfur coal desulfurization [3,4]. Consequently, desulfurization becomes indispensable for ensuring the high-quality and high-value utilization of liquefied residue. The intricate macromolecular structure of coal poses challenges in the complete removal of organic sulfur. Sulfur in coal predominantly exists in two forms: inorganic sulfur and organic sulfur. Beyond thiophene and its derivatives, sulfur manifests in inorganic forms such as sulfides (e.g., pyrite, marcasite) and sulfates (gypsum, baryte) [3,4,5,6]. The primary manifestation of inorganic sulfur is in the form of pyrite, originating from the reaction between iron and sulfide. Organic sulfur compounds present in the extractable organic matter of coal (bitumen) include thiols, sulfides, various thiophenes, and sulfur containing polycyclic aromatic compounds [7]. Sulfur is also present in coal in the complex macromolecular structure of kerogen (organic matter insoluble in conventional inorganic and organic solvents), which represents the dominant form of organic matter in coal. The removal of organic sulfur from the aliphatic carbon chains, and especially from the aromatic moieties, is a formidable task [3]. Generally, the proportion of inorganic sulfur is modest compared to organic sulfur. Currently, suitable desulfurization technologies for coal are categorized into physical desulfurization [8], chemical desulfurization [9], and biological desulfurization [10].

Diverse desulfurization methods, encompassing calcination [11], medium gas treatment, chemical desulfurization [4,12], microbiological treatment [13], and microwave techniques [14], have been devised by researchers. The dense flake form of coal restricts the application of physical desulfurization technologies like flotation technology [15]. Therefore, flotation exhibits limitations and removes only a portion of the inorganic sulfur, leaving organic sulfur unaddressed. Biological desulphurization poses challenges due to harsh conditions, making chemical methods more suitable for thorough desulphurization. Despite advancements in pre-combustion desulfurization, a method applicable to industrial-scale production remains elusive. Current research employs the electrochemical desulfurization method, renowned for its mildness [16], environmental friendliness [17], and efficiency [18]. Its versatility extends to gaseous [17], liquid fuel [19], and solid ore fuels [20].

Sulfur undergoes gaseous phase separation, necessitating subsequent steps for gas-phase sulfur removal and separation, thereby escalating application costs. Organic sulfur is amenable to chemical removal through thermochemical oxidation, converting it to sulfur-containing gases such as SO_2_ and SO_3_, or by electrochemical reduction to H_2_S and S^2−^ [21,22], and electrochemical oxidation, transforming organic sulfur into soluble sulfonic acid and sulfate [23]. However, the thermochemical oxidation desulfurization method is hampered by a lengthy process route, complex operating conditions, increased economic burden from sulfur-containing gas separation, and alterations of the coal organic matter due to high-temperature conditions.

Gong et al. investigated the impact of ionic liquid on coal water slurry desulfurization under a constant current HCl system, revealing that C_8_H_15_N_2_Br–HCl outperformed C_8_H_15_N_2_Cl–HCl in terms of a desulfurization effect [13]. Fan et al. showed that electrolytic oxidation can desulfurize the coal liquefaction residue under an alkaline condition [2]. Electrochemical reduction desulfurization, utilizing reducing agents like H_2_ and NaBH_4_, presents challenges, including the generation of H_2_S, a toxic and corrosive gas causing environmental pollution [24,25], and relatively high desulfurization costs. By contrast, electrochemical oxidation desulfurization offers mild reaction conditions, simple operation [24,26], facile separation of sulfur conversion products [27], and minimal impact on coal/coal liquefaction residue characteristics. Electrochemical oxidation desulphurization, commonly conducted in a slurry [28,29], demonstrates heightened effectiveness with an alkaline electrolyte [30,31], which is less detrimental to electrode materials. Electrochemical desulfurization under an alkaline environment, incorporating both alkali leaching [28] and electrochemical oxidation [32], enhances the removal of inorganic sulfur and organic sulfur in coal/coal liquefaction residue. The anode-focused alkaline system electrolytic oxidation desulfurization produces oxidation groups (ROS), serving as robust oxidants. Inorganic sulfur is oxidized to soluble sulfate [33,34], while organic sulfur undergoes oxidation to sulfoxide, further oxidizing to sulfone, and subsequent hydrolysis yields soluble sulfonic acid and sulfate. Filtration effectively removes soluble sulfate and sulfonic acid. Notably, sulfur in coal is predominantly oxidized to SO_4_^2−^ in the alkaline system, achieving desulfurization, while high purity H_2_ is generated at the cathode as a by-product [27], showcasing the broad applicability of the method.

To further illustrate the complex chemical and electrochemical processes involved in the desulfurization of coal, detailed reactions are provided here. The electrolytic desulfurization in alkaline systems includes several key steps. Inorganic sulfur, primarily as FeS_2_, undergoes transformations as follows [35]:

Inorganic sulfur (mainly pyrite, FeS_2_):

FeS_2_ + 9HO• → Fe(OH)_3_ + S_2_O_3_^2−^ + 3H_2_O + 2e^−^ (1)

2FeS_2_ + 3O_2_^–^ → 2Fe^3+^ + 2S_2_O_3_^2−^ + 5 e^−^ (2)

Organic sulfur:

O_2_ + 2R–S–S–R → 2R–S–S(O)R (3)

2O_2_ + R–S–S–R → R–S(O_2_)–S(O_2_)R (4)

R–S(O_2_)–S(O_2_)R + 2H_2_O → R–OH + R–OH + 2SO_4_^2−^ (5)

In the acidic system, reactive oxygen species (ROS) are produced by the oxygen reduction reaction (ORR), a part of the electro-Fenton process. High-valence metal ions such as Fe, V, Mn, Co, Ni, Cu, etc. act as oxidants. The reactions involving these metal ions in an acidic system include the following [35]:

Fe^3+^ + nHSO_3_ ↔ Fe^3+^(HSO_3_)n (6)

Fe^3+^ + nHSO_3_ ⇄ Fe^2+^ + (n−1)HSO + H^+^ + SO_3_^−^ (7)

Fe^3+^ + SO_3_^−^ + H_2_O ↔ Fe^2+^ + HSO_4_^−^ + H^+^ (8)

SO_3_^−^ + O_2_ ⇄ SO_5_^−^ (9)

(10) $${\mathrm{Fe}}^{2+}+{\mathrm{SO}}_{5^{-}}\overset{H^{+}}{\to }{\mathrm{Fe}}^{3+}+{\mathrm{HSO}}_{5^{-}}$$

Fe^2+^ + HSO_5_^−^ → Fe^3+^ + SO_4_^2–^ + OH^−^ (11)

(12) $${\mathrm{Fe}}^{2+}+{{\mathrm{SO}}_{4}}^{2-}\;\overset{H^{+}}{\to }{\mathrm{Fe}}^{3+}+{\mathrm{HSO}}_{4^{-}}$$

High-efficiency catalysts can promote the formation of oxidants, ensuring the complete oxidation of sulfur. Nonetheless, the electrode design minimally impacts the full separation of sulfur from minerals. Integrating electro-oxidation with other techniques enhances this separation after thorough oxidation. For instance, combining electro-oxidation with distillation achieves over 98% sulfur removal in gasoline, compared to just 0.82% removal with direct distillation alone [36]. When electro-oxidation is paired with solvent extraction, sulfur levels in condensed gasoline decrease significantly, and the transformed organic sulfur can be effectively isolated using polar solvents [37]. Unlike ROS, valence ions exhibit longer lifespans and are less susceptible to quenching, which enhances their oxidation effectiveness in acidic environments. However, these ions can form precipitates and engage in redox competition with sulfur, potentially leading to secondary fuel pollution. For example, Cl^−^ from hydrochloric acid can react with organic matter associated with (bounded to) minerals to form difficult-to-remove organic chlorine groups, contributing to secondary pollution [35]. A common challenge in both electro-reduction and electro-oxidation desulfurization is enhancing the interaction between the medium and reactants.

The hydroxide radicals generated in the solution react with the absorbed oxygen on the electrode surface, forming high-valence oxides through the following reactions [35]:

MO_x_ + H_2_O → MO_x_(OH) + H^+^ + e^−^ (13)

MO_x_ + HO^–^ → MO_x_(OH) + e^−^ (14)

The absorbed HO• reacts with the oxygen molecules on the electrode surface and transfers oxygen into metal oxide to form a high-valence oxide [35].

MO_x_(OH) → MO_x+1_ + H^+^ + e^−^ (15)

MO_x_(OH) + HO^–^ → MO_x+1_ + H_2_O + e^−^ (16)

The ROS oxidizes the substance in the solution.

MO_x_(OH)_y_ + yR → MO_x_ + yH^+^ + ye^−^ + yRO (17)

MO_x+1_ + R → MO_x_ + RO (18)

The critical role of mass transfer in direct oxidation processes is highlighted, as it is the rate-controlling step when other parameters such as electrode materials and electrolysis conditions are optimized. Unlike direct oxidation, indirect oxidation is more commonly recognized as the dominant process in electrolytic desulfurization, converting two-dimensional oxidation into three-dimensional bulk oxidation, thus enhancing the collision between sulfur-containing phases and the ROS.

In both acidic and alkaline systems, HO• is generated through several steps:

Acidic system

O_2_ + H^+^ + e^−^ → OOH• (19)

OOH• + H^+^ + e^−^ → O• + H_2_O (20)

O• + H^+^ + e^−^ → HO• (21)

HO• + H^+^ + e^−^ → H_2_O (22)

Alkaline system

O_2_ + H_2_O + e^−^ → OOH• +OH^–^ (23)

OOH• + e^−^ → O• + OH^–^ (24)

O•+H_2_O + e^−^ → HO• + OH^–^ (25)

HO• + e^−^ → OH^–^ (26)

These equations collectively describe the complex interplay of chemical reactions occurring during the electrolytic desulfurization of coal, underlining the intricate mechanisms that contribute to the efficient removal of sulfur. These reactions elucidate the pathways by which sulfur compounds are decomposed in both acidic and alkaline environments to achieve desulfurization.

Electrochemical desulfurization research has yielded promising results in the removal of organic and inorganic sulfur from coal. However, there is a notable dearth of reports on coal desulfurization, especially using acidic organic media with implications for the electrochemical method, and an insufficient exploration of the impact on coal quality and reaction mechanisms. Despite advancements in new energy power generation technologies enabling the use of inexpensive renewable electricity for desulfurization, the energy consumption of the electrochemical methods remains a concern. This paper addresses this by emphasizing the significance of basic data related to energy consumption for the widespread application of electrochemical oxidation and desulfurization. The relationship between desulfurization energy consumption and efficiency, often overlooked in many studies, is pivotal. This study introduces an efficiency index, the energy consumption per unit mass of sulfur removal, and explores its association with desulfurization efficiency [37].

## 2. Materials and Methods

The subbituminous coal sample was collected from the Bogovina East field (Lower Miocene, approximately 20–16 million years ago) in the Bogovina Basin, Eastern Serbia. According to an average huminite reflectance, Rr, of 0.42 ± 0.04% [38], this coal is classified as bright brown coal (low-rank A). The sample was selected based on previous studies of the Bogovina East Field [4,38,39], which indicated a high sulfur content, relatively high mineral matter, and a significant amount of liptinites, the most reactive maceral group. Thus, it serves as a good substrate for testing desulfurization efficiency.

The air-dried sample was crushed using a Fritsch Mortar Grinder Pulverisette 2 and sieved through a 200 µm sieve. Proximate analysis (determination of analytical moisture, volatile matter, ash, and fixed carbon) was conducted according to the ASTM D3172 standard [40], while total sulfur content was determined by the Eschka method [4]. The Bogovina coal sample used in this study contained 9.38 wt.% moisture, 55.09 wt.% volatile matter, 17.24 wt.% ash, 21.97 wt.% fixed carbon, and 6.91 wt.% total sulfur.

A two-electrode electrochemical cell arrangement was used for electrochemical desulfurization. The anodes were a graphite rod and a dimensionally stable titanium-based metal oxide (ruthenium-titanium oxide) coated anode (DSA), both with an active surface of 70 cm^2^. A 15% TiCl_3_ solution in 10% aqueous HCl (Sigma Aldrich, Taufkirchen, Germany) and RuCl_3_ (cryst.) (Sigma Aldrich, Taufkirchen, Germany) were utilized to prepare the corresponding sols via condensation and forced hydrolysis, with simultaneous oxidation (aging) in air at elevated temperatures, following a procedure described elsewhere [41]. The TiCl_3_ solution was added to a highly acidic, stirred, boiling HCl solution (6 M concentration) and maintained at the boiling point for 12 h. Additionally, TiO_2_ sol was obtained from a TiCl_3_ solution that had been pre-oxidized by adding 30% aqueous H_2_O_2_, until a color change from violet to yellow was observed [42]. In both experiments, the TiCl_3_ solution was added to achieve a dispersion with approximately 0.8% mass of the solid phase.

The RuO_2_ sol was prepared by adding solid RuCl_3_ to boiling, stirred water, resulting in a dispersion with about 1% mass of the solid phase. This solution was also kept at a boiling temperature for 7 h. The resulting TiO_2_ and RuO_2_ sols, as well as their mixtures, remained as stable dispersions for over 3 months. Separate dispersions of TiO_2_ and RuO_2_, as well as their mixtures (with a Ti:Ru ratio of 60:40), were evaporated.

To fabricate an anode, the sol mixture, previously concentrated by evaporation to reach approximately 0.4% mass of the solid phase, was applied to a titanium hollow cylinder. The titanium surface was pre-treated according to the recommended thermal procedure for RuO_2_-TiO_2_ coating formation, including sandblasting, pickling with hot HCl solution, and degreasing by dipping into a NaOH–ethanol mixture. The Ti substrate was coated using a multilayer painting process with the RuO_2_–TiO_2_ sol mixture. After each coating step, the layer was calcined at 450 °C for 10 min. This procedure was repeated until a coating of 2 mg cm^−2^ was achieved. Finally, the samples were calcined at 450 °C for 3 h.

A 316L stainless steel cylinder with an active surface of 280 cm^2^ was used as the cathode. Suspensions were prepared by adding 60 g/L of the crushed and sieved coal sample to a 0.1 M HCl solution (Sigma Aldrich, Taufkirchen, Germany). The electrochemical cell was filled with the suspension, and an Aim and Thurlby Thandar Instruments TTi CPX400DP Bench/System/ATE Programmable DC power supply (Aim and Thurlby Thandar Instruments, Cambridgeshire, United Kingdom) was used. The suspensions were continuously stirred by a magnetic stirrer MS-500D (Witeg Labortechnik, Wertheim, Germany) during electrochemical desulfurization. Measurements were carried out for 4 h at 30, 40, 50, 60, and 70 °C. After each experiment, the coal was filtered from the supernatant, dried, and analyzed for sulfur content, while the supernatant was used in further experiments.

The electrochemical properties of the supernatant were studied using cyclic voltammetry (CV) and linear sweep voltammetry (LSV). The electrochemical measurements were performed in a conventional three-electrode cell. A platinum wire and Ag|AgCl were used as the counter and reference electrodes, respectively. All potentials are referenced to Ag|AgCl. The working electrode was glassy carbon (GC, Sigradur–Sigri, Elektrographite, GmbH, Germany) with a surface area of 0.20 cm^2^. The cell was purged with N_2_ for 30 min prior to CV and LSV measurements. A potentiostat/galvanostat measuring station (BioLogic SAS, SP-240, Grenoble, France) with physical electrochemistry software was used. CV responses in 0.10 M HCl were recorded at a scan rate of 50 mV s^−1^, while LSV responses were recorded at a scan rate of 2 mV s^−1^.

Copper deposition was performed in a two-electrode electrochemical cell, using a DSA with an active surface of 70 cm^2^ as the anode and a 316L stainless steel cylinder with an active surface of 280 cm^2^ as the cathode. The supernatant was used for the Cu recovery by filling the electrochemical cell, and an Aim and Thurlby Thandar Instruments TTi CPX400DP Bench/System/ATE Programmable DC power supply was used. The suspensions were continuously stirred by a magnetic stirrer during the process.

The concentration of metals (total, in all oxidation states present) was measured using inductively coupled plasma optical emission spectrometry (ICP-OES). ICP-OES measurements were performed on an iCAP 6500 Duo ICP instrument (Thermo Fisher Scientific, Cambridge, UK) with iTEVA operating software (ver. 9.9.2). Samples were introduced into the plasma by direct liquid aspiration. Calibration standard solutions in the appropriate concentration range (1–50,000 µg L^−1^) were prepared from a certified standard solution: Plasma standard solutions, Specpure^®^ (Alfa Aesar GmbH & Co KG, Emmerich am Rhein, Germany), were used. The correlation coefficients for metals were >0.99. The concentration measurements were repeated three times (*n* = 3) with a relative standard deviation of RSD < 0.5%.

## 3. Results

### 3.1. Electrochemical Desulfurization of Coal

A comprehensive analysis of the electrochemical desulfurization process for subbituminous coal from the Bogovina basin was conducted using both graphite and dimensionally stable anode (DSA) electrodes. The primary focus was on the assessment of activation energy, desulfurization rate, total energy consumption, energy consumed per kilogram of sulfur removed, and the efficiency of energy use in relation to the increase in the desulfurization rate.

**Figure 1:**
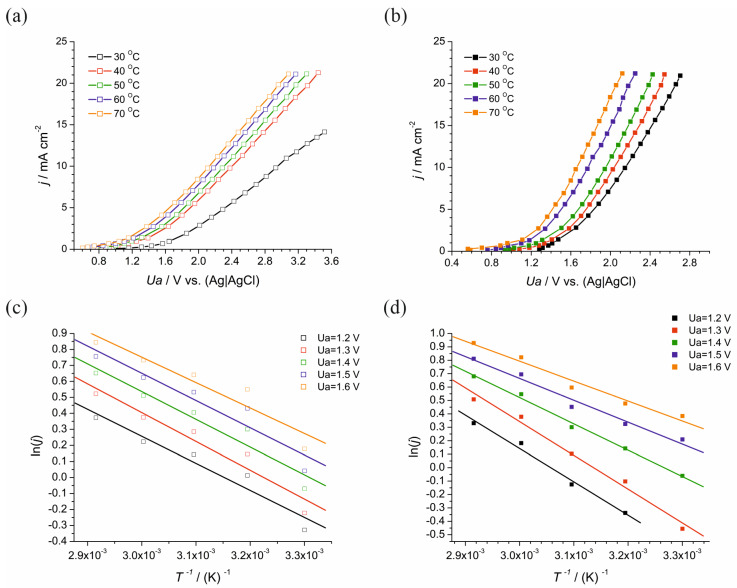
(**a**) LSV polarization curves for graphite anode and (**b**) LSV polarization curves for DSA anode at various temperatures. Arrhenius plots for (**c**) graphite and (**d**) DSA anodes for different anodic potentials.

Figure 1a,b presents the LSV polarization curves for the graphite anode and the DSA. It displays the variation in current density with applied anodic potential over the temperature range of 30–70 °C. The temperature-dependent increase in current density indicates an enhanced reaction rate with elevated temperatures, suggesting an Arrhenius-type behavior where the reaction kinetics is accelerated by thermal energy. Figure 1c,d illustrates the Arrhenius plots for the graphite anode and the DSA, respectively, at different applied anodic potentials. These plots represent the natural logarithm of the current density (ln(*j*)) as a function of the reciprocal absolute temperature (1/*T*). The slope of these lines is related to the activation energy (*E*a) of the desulfurization process.

**Figure 2:**
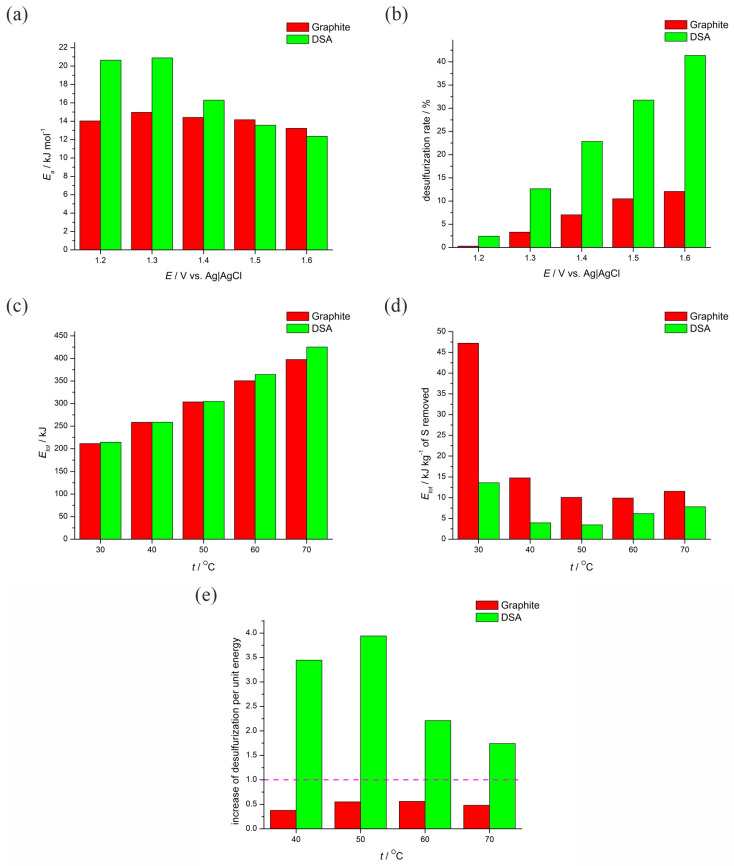
(**a**) Activation energy for graphite and DSA electrodes at various anode potentials, (**b**) desulfurization rate for graphite and DSA electrodes at various anode potentials, (**c**) total energy consumption and requirements for graphite and DSA anodes at various temperatures, (**d**) energy requirements for removal of kilogram of sulfur, and (**e**) increase of desulfurization per unit of energy.

The desulfurization performance of the two electrodes (a commonly used graphite anode and a DSA) was further evaluated through various efficiency metrics. Figure 2a highlights the activation energy (*E*a) requirements for the desulfurization process at different potentials. A lower activation energy indicates that the electrode facilitates the desulfurization reaction at a lower energetic cost.

Figure 2b shows the desulfurization rate at various anode potentials for both electrodes, where higher rates correspond to a more efficient desulfurization process. Figure 2c documents the total energy consumption and energy requirements for the desulfurization process across various temperatures and a current density of 2.5 mA cm^−2^, reflecting the overall energy demands of the process; while Figure 2d delineates the specific energy consumption at 2.5 mA cm^−2^, indicating the energy consumed per kilogram of sulfur removed from the coal. Lower values in this metric signal imply a more energy-efficient process. Figure 2e captures the increase in desulfurization per unit of energy used, providing insight into the process efficiency. Values above one (purple line on the graph) suggest that the desulfurization efficiency is improving faster than the rate of energy consumption. The data demonstrates the justification for the increase in temperature and the corresponding rise in energy consumption. Specifically, if the ratio is above one, the increase is warranted.

### 3.2. Influence of Electrochemical Desulfurization on Coal Composition

The proximate analysis of untreated coal and coal samples subjected to desulfurization using the dimensionally stable anode (DSA) and the graphite anode at 50 °C for 4 h is presented in Table 1.

### 3.3. Concentrations of Metals in Supernatant Obtained by Electrochemical Desulfurization of Coal

The most efficient electrochemical desulfurization of Bogovina Basin coal was conducted at 50 °C for 4 h. The concentration of metals in the supernatant after this desulfurization experiments are presented in Table 2.

**Figure 3:**
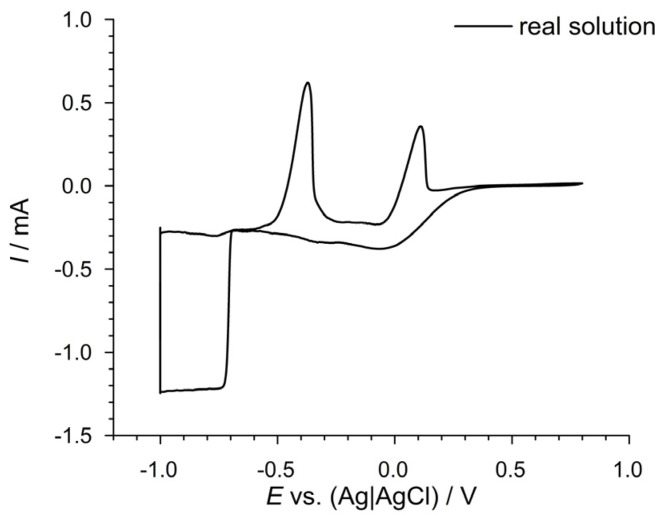
Cyclic voltammogram of the supernatant solution obtained from the electrochemical desulfurization of Bogovina Basin coal.

After removal of the desulfurized coal, the supernatant solution was investigated for the recovery of copper (Cu) at 25 °C. The cyclic voltammogram (CV) of the supernatant solution, displayed in Figure 3, reveals distinct peaks corresponding to the reduction and oxidation processes of the metal ions present.

The main obstacles identified in the recovery process were the presence of lead (Pb) and iron (Fe). Lead was found to significantly affect the composition of the solution, while the presence of iron influenced the efficacy of the electrochemical process due to the redox reactions.

**Figure 4:**
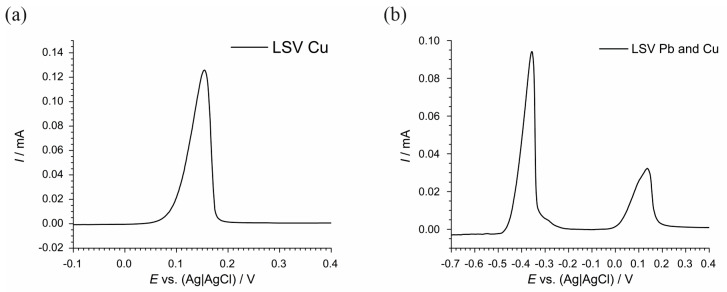
Linear sweep voltammograms (LSVs) of laboratory solutions containing (**a**) only copper (Cu) and (**b**) copper (Cu) and lead (Pb) after 5 min hold at −1.00 V.

To investigate further, solutions containing only Cu (Figure 4a) and both Cu and Pb (Figure 4b) were prepared and tested with concentrations equal to those found in the real system (Table 2). Both LSVs were performed after a 5-min hold at −1.00 V to ensure a complete reduction of all species. The potential values read from the figures show that a distinct oxidation peak is observed at approximately 0.15 V, indicating the oxidation of Cu^0^ back to Cu^2+^ for the solution containing only Cu (Figure 4a); while, for the solution containing both Cu and Pb (Figure 4b), the LSV indicates that the presence of Pb does not significantly hinder the recovery of Cu, as evidenced by the oxidation peak of Cu appearing at approximately 0.15 V.

**Figure 5:**
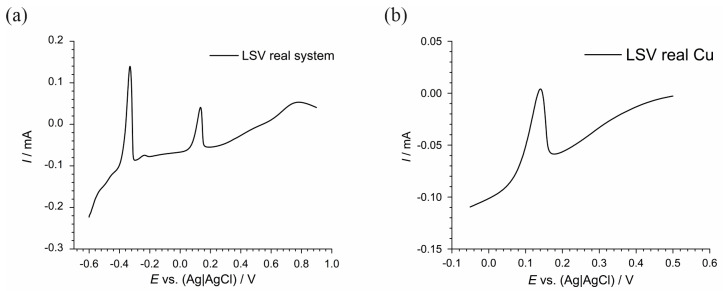
Linear sweep voltammograms (LSVs) of the real supernatant solution obtained from the electrochemical desulfurization of the Bogovina coal. (**a**) LSV performed from −0.70 V after 5 min hold at −1.00 V and (**b**) LSV performed after holding the potential at −0.15 V for 5 min to allow Cu to be deposited on the cathode.

To investigate the selective recovery of Cu, linear sweep voltammograms (LSVs) were performed on the real supernatant solution obtained from the electrochemical desulfurization of the Bogovina coal. Figure 5a shows the LSV performed from −0.70 V after a 5-min hold at −1.00 V to ensure the complete reduction of all species, indicating that it is possible to remove only Cu from the system. This is evidenced by the reduction peak corresponding to the Cu^2+^/Cu^0^ redox couple.

Subsequently, the potential was held at −0.10 V for 5 min to allow Cu to be deposited on the cathode. The LSV in Figure 5b shows a distinguishable Cu dissolution peak at approximately 0.15 V during the anodic sweep, confirming the successful deposition and subsequent oxidation of Cu.

## 4. Discussion

### 4.1. Efficiency of Electrochemical Desulfurization of Coal

The LSV polarization curves (Figure 1a,b) demonstrate a pronounced temperature effect, where increased thermal energy translates into higher current densities, reflective of the Arrhenius behavior of chemical reactions. While this is true for both electrodes, the DSA electrode exhibits a steeper slope, indicating a more pronounced response to temperature changes. This response suggests that, at higher operational temperatures, the DSA electrode may sustain elevated reaction rates without a commensurate increase in energy consumption, thereby enhancing the desulfurization rate while maintaining energy efficiency. The curves depict a significant increase in current density over the same temperature range, indicating that the DSA electrode catalyzes the desulfurization reaction more effectively than the graphite electrode. The Arrhenius plots (Figure 1c,d) reveal a lower activation energy for the DSA electrode at higher potentials, implying a reduction in the intrinsic energy barrier for desulfurization. This reduction is not merely a reflection of thermodynamic favorability but also translates into enhanced kinetic proficiency. The slope of the lines in the Arrhenius plots is directly related to the activation energy of the desulfurization process. A steeper slope corresponds to higher activation energy.

The activation energy is a crucial parameter, as it indicates the energy barrier that must be overcome for the desulfurization reaction to proceed (Figure 2a). A lower activation energy signifies that the electrode facilitates the reaction more efficiently, requiring less energy input. As can be seen in Figure 2a, the activation energy for the DSA is generally higher at lower potentials (1.20 V to 1.40 V) compared to the graphite anode. This indicates that, at these potentials, the graphite anode should be more effective in reducing the energy barrier for the desulfurization reaction. At higher potentials (1.50 V and 1.60 V), the activation energy for both anodes decreases and becomes more comparable, suggesting that the energetic cost of the reaction is less dependent on the anode material at these higher potentials. The variation in activation energy with different anode materials and applied potentials has significant implications for the overall efficiency of the desulfurization process. The desulfurization rate (Figure 2b) is a critical measure of the process’s efficiency, with higher rates indicating more effective sulfur removal from the studied coal. At 1.20 V, both electrodes exhibit very low desulfurization rates, with the DSA showing a marginally higher rate compared to graphite. At 1.30 V, the DSA significantly outperforms the graphite anode, achieving a desulfurization rate of approximately 12%, while the graphite anode achieves less than 5%. This indicates that at lower potentials, the DSA is more effective in facilitating the desulfurization reaction. At 1.40 V, the desulfurization rate for the DSA increases further, reaching about 20%, while the graphite anode shows a desulfurization rate of approximately 10%. The efficiency gap between the two electrodes remains significant. At 1.50 V, the DSA achieves a desulfurization rate of approximately 30%, whereas the graphite anode’s rate increases to about 15%. Therefore, the DSA continues to demonstrate superior performance. At 1.60 V, the DSA shows a substantial desulfurization rate of approximately 40%, whereas the graphite anode’s rate is approximately 20%. This indicates that the DSA is twice as effective as the graphite anode at this potential. The DSA consistently shows higher desulfurization rates across all tested potentials, indicating superior catalytic properties for the desulfurization reaction. This suggests that DSAs are more efficient in promoting sulfur removal, likely due to better electrochemical characteristics and surface properties. The optimal potential for maximizing the desulfurization rate with the DSA appears to be approximately 1.60 V, where the highest rate is achieved. For the graphite anode, while the desulfurization rate improves with increasing potential, it does not match the performance of the DSA at any tested potential. Higher desulfurization rates translate to more effective sulfur removal per unit time, potentially reducing the overall energy consumption and cost of the desulfurization process. The use of DSAs, despite possibly higher initial costs, could result in long-term savings due to their higher efficiency.

Figure 2c,d offers a comprehensive view of the energy demands and efficiency of the electrochemical desulfurization process using graphite anodes and DSAs. The charts show total energy consumption and energy consumption per kilogram of sulfur removed at a current density of 2.5 mA cm^−2^ across various temperatures. Both anodes show an increase in total energy consumption with temperatures rising from 30 to 70 °C. At 30 and 40 °C, the total energy consumption for graphite anodes and DSAs is relatively similar, indicating comparable energy demands at these lower temperatures. At higher temperatures (50–70 °C), the DSA tends to consume slightly more energy than the graphite anode, especially at 70 °C where the energy consumption difference is more pronounced. The graphite anode demonstrates a more consistent increase in energy consumption across the temperature range. The DSA, while showing higher energy consumption at elevated temperatures, might be balancing this with higher desulfurization rates, as seen in the previous graphs. Lower values in this metric are desirable for specific energy consumption per kilogram of sulfur removed (Figure 2d) as they signal a more energy-efficient desulfurization process. At 30 °C, the specific energy consumption for the graphite anode is significantly higher than that of the DSA anode, indicating that the DSA is much more efficient at this lower temperature. As the temperature increases, the specific energy consumption for both anodes decreases, reflecting an enhanced process efficiency at higher temperatures. From 40 to 70 °C, the DSA consistently shows lower specific energy consumption compared to the graphite anode. This demonstrates that the DSA not only facilitates a higher desulfurization rate but does so more efficiently in terms of energy usage per unit of sulfur removed. The gap in specific energy consumption between the two anodes narrows at higher temperatures, suggesting that while the DSA is more efficient at lower temperatures, both anodes perform similarly at higher temperatures.

Figure 2e shows the incremental efficiency of the desulfurization process for graphite anodes and DSAs across various temperatures. Incremental efficiency is a measure of how effectively energy is utilized to achieve desulfurization. Higher values indicate a more efficient use of energy, meaning more sulfur is removed per unit of energy consumed. The incremental efficiency is plotted against temperature, with a reference line at y = 1. Values above this line indicate that increasing the temperature (and thus energy consumption) results in a proportionally higher increase in sulfur removal, making the energy expenditure justified. For temperatures of 40 and 50 °C, the DSA shows significantly higher incremental efficiency compared to the graphite anode. The values for the DSA are well above one, indicating that at these temperatures, increasing energy consumption is highly effective in enhancing desulfurization. At 60 and 70 °C, the incremental efficiency for the DSA decreases but remains above one, suggesting continued effectiveness, albeit with diminishing returns compared to lower temperatures. For the graphite anode, the incremental efficiency remains below one across all temperatures, indicating less effective energy use. The DSA consistently outperforms the graphite anode in terms of incremental efficiency at all temperatures.

A critical aspect of the analysis is the determination of the optimal operational temperature that harmonizes the competing demands of reaction kinetics and energy efficiency. The data indicate that 50 °C is the pivotal temperature for the DSA electrode, where it achieves a notable balance between the desulfurization rate and energy consumption. The LSV polarization curves (Figure 1a,b) and activation energy plots (Figure 1c,d) converge on this temperature as a point of inflection where the reactivity significantly improves while maintaining an economically viable energy input. At 50 °C, the specific energy consumption reaches its lowest value for the DSA electrode, as shown in Figure 2d, implying the most sulfur removal for the least energy expenditure. When all performance metrics are synthesized, 50 °C emerges as the optimal temperature from an integrated perspective. This optimum reflects a temperature regime where the DSA electrode’s desulfurization rate is near its peak efficiency while also retaining a high increase in desulfurization per unit energy, as delineated by Figure 2e. At temperatures below 50 °C, although the energy consumption per kilogram of sulfur removed is relatively lower, the desulfurization rates are not maximized, thus prolonging the process duration. Conversely, at temperatures above 50 °C, the desulfurization rate continues to increase; however, this is at the expense of significantly higher energy consumption, as reflected by the increase in both total energy consumption (Figure 2c) and the activation energy required at elevated potentials (Figure 2a). The incremental efficiency metric (Figure 2e) is particularly insightful, revealing that at 50 °C, the desulfurization efficiency gains outpace the energy input increments. Thus, while the energy consumption does rise with temperature, it does so in a manner that is disproportionately beneficial to the desulfurization outcome at this specific operational point. Beyond 50 °C, there is an evident diminishing return where increased energy consumption does not equate to proportional desulfurization benefits, marking 50 °C as the threshold for maximizing process efficiency.

### 4.2. Changes in Coal Composition

The proximate analysis results of untreated coal and coal samples subjected to desulfurization using a dimensionally stable anode (DSA) and a graphite anode at 50 °C for 4 h, as presented in Table 1, reveal notable changes in the composition of the coal samples. Untreated coal exhibited a high moisture content of 9.38%, which significantly decreased upon treatment with both desulfurization methods. The sulfur content in the untreated coal was 6.96%. After desulfurization, the sulfur content decreased to 6.12% for the graphite anode treated sample (12.0% mass reduction) and to 4.08% for the DSA-treated sample (41.4% mass reduction). This indicates that the DSA method was more effective in reducing sulfur content compared to the graphite anode method. Ash content also saw a significant reduction in both treated samples. The untreated coal had an ash content of 16.39%, which was reduced to 8.25% in the graphite anode-treated sample and was further reduced to 4.35% in the DSA-treated sample. This suggests that the DSA method is more efficient in reducing ash content. The volatile matter content in untreated coal was 52.38%. After treatment, the graphite anode-treated sample showed a slight increase in volatile matter content to 53.24%, while the DSA-treated sample showed a decrease to 49.86%. The increase in volatile matter content in the graphite-treated sample may indicate a partial restructuring of the coal matrix, whereas the decrease in the DSA-treated sample suggests a different reaction pathway. Finally, the fixed carbon content (C_fix_) in untreated coal was 21.84%. This value increased to 37.73% in the graphite anode-treated sample and to 44.86% in the DSA-treated sample. The substantial increase in fixed carbon content in both treated samples, particularly in the DSA-treated sample, indicates the considerable removal of mineral matter. This has a positive impact on calorific value, and both desulfurization methods effectively enhance the coal’s fixed carbon content, with the DSA method being more effective.

### 4.3. Copper Recovery from the Supernatant

After removing the coal, the supernatant solution was investigated for the recovery of copper (Cu) at 25 °C. The cyclic voltammogram (CV) of the supernatant solution, showed in Figure 3, reveals distinct peaks corresponding to the reduction and oxidation processes of the metal ions present. The potential was held at −1.00 V for 5 min to ensure the complete reduction of all species. The CV profile indicates the presence of copper, along with other metal ions such as lead (Pb) and iron (Fe). The initial cathodic sweep shows reduction peaks at approximately −0.370 V vs. (Ag|AgCl), which corresponds to Pb^2+^ + 2*e*^−^ ⇌ Pb (*s*), *E°* = −0.126 V vs. standard hydrogen electrode (SHE), that can be attributed to the reduction of Pb^2+^ to Pb^0^, and at 0.150 V vs. (Ag|AgCl), which corresponds to Cu^2+^ + 2*e*^−^ ⇌ Cu (*s*), *E°* = 0.337 V vs. SHE, that can be attributed to the reduction of Cu^2+^ to Cu^0^. The subsequent anodic sweep reveals a broad oxidation peak at approximately 0.00 V, indicating the oxidation of Cu^0^ back to Cu^2+^. These observations confirm the feasibility of recovering copper from the supernatant solution post-desulfurization. The primary challenges encountered in the recovery process were the presence of lead (Pb) and iron (Fe). Lead significantly altered the solution’s composition, while iron affected the efficiency of the electrochemical process due to its involvement in redox reactions:

Fe^3+^ + e^−^ ↔ Fe^2+^ (E^0^ = 0.771 V vs. SHE) (27)

Fe_2_O_3_ (s) + 6 H^+^ + 2 e^−^ ↔ 2 Fe^2+^ +3 H_2_O (E^0^ = 0.728 V vs. SHE) (28)

Figure 4a presents the LSV for a prepared solution containing copper (Cu), while Figure 4b presents the LSV for a prepared solution containing both copper (Cu) and lead (Pb), which were tested to investigate the feasibility of recovering Cu from a complex solution post-electrochemical desulfurization. Figure 4a displays the LSV for a solution containing only Cu. The voltammogram reveals a prominent oxidation peak at approximately 0.15 V (vs. Ag|AgCl), which is indicative of the oxidation of Cu^0^ back to Cu^2+^. This clear peak demonstrates that Cu can be effectively deposited and subsequently oxidized under the experimental conditions, validating the method for the Cu recovery in a simplified system without interfering metal ions. Figure 4b illustrates the LSV for a solution containing both Pb and Cu, with concentrations equivalent to those found in the real system. In this voltammogram, a significant oxidation peak is observed at approximately 0.15 V (vs. Ag|AgCl), which is consistent with the oxidation of deposited Cu. This result suggests that despite the presence of Pb, Cu can still be selectively deposited and recovered. However, the voltammogram shows additional features that are attributed to the presence of Pb, indicating its influence on the electrochemical behavior of the system. These results are crucial as they show that in the presence of Pb, the recovery of Cu remains feasible, albeit with some interference. The presence of Pb does not preclude the deposition and oxidation of Cu, though careful control of the electrochemical parameters is necessary to optimize the recovery process. This investigation supports the potential for selectively recovering Cu from a real, complex solution resulting from the desulfurization process, highlighting the importance of understanding and managing the influences of co-existing metal ions like Pb in the recovery system.

The LSV of the supernatant solution obtained from the electrochemical desulfurization of the Bogovina coal starting from −0.70 V after initial hold at −1.00 V is shown in Figure 5a. Figure 5b depicts the LSV of the real system after holding the potential at −0.15 V for 5 min to deposit Cu on the cathode. These figures were analyzed to evaluate the feasibility of selectively recovering copper from a complex mixture of metal ions. The first LSV demonstrates the possible reduction processes occurring within the solution. The significant peaks observed in the voltammogram are at approximately −0.34 V and 0.15 V. The peak at −0.34 V corresponds to the oxidation of Pb^0^ to Pb^2+^, indicating the presence of lead in the solution. The peak at 0.15 V corresponds to the oxidation of Cu^0^ back to Cu^2+^, suggesting that copper can be selectively recovered from the system through controlled electrochemical processes. This distinct peak confirms that Cu was successfully deposited on the cathode during the holding period and could be subsequently oxidized, providing evidence that Cu can be selectively recovered from the real solution. Based on the observations from Figure 5b, it was decided to perform the deposition (potential hold) at a potential of −0.10 V. This decision was driven by the need to optimize the conditions for Cu deposition and minimize interference from other metal ions present in the solution. The results indicate that holding at −0.15 V should allow for effective deposition of Cu, facilitating its subsequent recovery through oxidation processes. These findings highlight the potential for selective Cu recovery from the complex solution resulting from coal desulfurization. By carefully controlling the electrochemical conditions, particularly the deposition potential, Cu was efficiently separated and recovered, demonstrating the viability of this method for treating such complex mixtures.

In our analysis, we opted to conduct the copper recovery at a potential of −0.15 V vs. (Ag|AgCl). Over the course of the 4-h copper recovery period, the current was observed to shift from −4.6 mA to −5.2 mA, reflecting the dynamic changes in the electrochemical conditions during the deposition process. During these 4 h of copper deposition on the austenitic steel electrode, which had an area of 270 cm^2^, the process was carried out using 0.8 L of the real solution. As a result, 0.79 mg of copper was successfully recovered. This adjustment in the operational voltage was strategically chosen to optimize the recovery efficiency of copper under the given experimental conditions.

The high concentration of Fe in the system poses a significant challenge due to its competing reactions, which consume part of the applied current, thereby reducing the efficiency of the Cu recovery. The actual recovery efficiency of 86.34% (0.79 mg out of 0.91 mg available in the solution was recovered) indicates that the process is effective for recovering a substantial amount of Cu from the solution. This high recovery rate suggests that the conditions used in the experiment are close to optimal. Nevertheless, further optimization could potentially improve the recovery rate even more. The experimental results underscore the importance of precise control in electrochemical processes and the need for further investigation to enhance the recovery rates of valuable metals from complex solutions. Factors such as electrolyte composition, potential hold time, and the influence of other metal ions need to be carefully adjusted to improve the overall efficiency.

To calculate the energy consumption for the Cu recovery process, we need to determine the total electrical energy used during the electrochemical process. The energy consumption for our process can be calculated as follows:

*E* = *V* × *I*_avg_ × *τ* (29)

where *V* is the applied potential (V), *I*_avg_ is the average current of the process (A) and *τ* is the time (s).

(30) $$I_{avg}=\frac{I_{initial}+I_{final}}{2}$$

When we take that *V* = 0.15 V, *I*_initial_ = −4.6 mA, *I*_final_ = −5.2 mA and *τ* = 4 h (14,400 s), we determine that *E* = 10.56 W s = 10.56 J.

The authors calculated the feasibility of recovering 1 t of Cu from the solution with the concentration given in Table 2, considering the average electricity price of 0.21 € kWh^−1^ and the copper price of 9000 € t^−1^, both taken from June 2024.

Energy consumption per unit of mass is calculated as follows:

(31) $$E_{m}=\frac{E}{m}$$

and the *E*_m_ = 13,410 J g^−1^, or 0.00372 kWh g^−1^. Hence the total cost of recovery of 1 t of Cu is 781.20 €. This price is significantly lower compared to other traditional copper recovery processes (recovery from mine tailings, hydrometallurgical and leaching processes). The recovery cost of copper can reach from around 2060 €/t [43] up to 2788 €/t [44]. It must be stated that not that much research or economic analyses have been conducted on valuable metal recovery from coal and coal combustion products and byproducts.

## 5. Conclusions

This study explored the efficacy of different anodes in the electrochemical desulfurization of coal, specifically comparing the performance of graphite and DSA electrodes. The DSA electrode demonstrated significantly higher desulfurization rates compared to the graphite anode due to its superior electrochemical properties, which enhance oxidation reactions necessary for sulfur removal. The DSA exhibited lower activation energy at higher potentials, making the desulfurization process more energy efficient. This translates to a reduced energy input for the same level of sulfur removal. Additionally, the DSA showed a more pronounced response to temperature increases, with the desulfurization rate improving significantly at higher temperatures. The optimal operational temperature for the DSA electrode was identified as 50 °C, achieving the best balance between desulfurization efficiency and energy consumption.

The electrochemical treatment significantly reduced the sulfur content in coal. This study identified −0.15 V vs. (Ag|AgCl) as an optimal potential for Cu deposition, allowing for the effective separation and recovery of copper with minimal interference from other metal ions like lead (Pb) and iron (Fe). This optimized potential, along with careful control of electrochemical parameters, resulted in a recovery efficiency of 86.34% for copper, which is close to optimal but still open to further improvement. The energy consumption for the recovery process was calculated to be 10.56 J, with the total cost of to recover 1 ton of Cu from the solution to be approximately 781.20 €. This demonstrates the economic feasibility of the process under the given experimental conditions and highlights the importance of energy-efficient recovery methods for valuable metals from complex solutions. However, the presence of high concentrations of Fe posed a challenge due to its competing reactions, which reduced the efficiency of the Cu recovery. This underscores the need for further optimization of electrochemical conditions and the management of co-existing metal ions to improve the overall efficiency and recovery rates.

## Figures and Tables

**Table 1:** Proximate analysis of untreated coal and coal after desulfurization with DSA and graphite anode after 4 h at 50 °C.

Sample	Moisture, %	S, %	Ash, %	Volatile Matter, %	C_fix_
Untreated coal	9.38	6.96	16.39	52.38	21.84
Graphite	0.78	6.12	8.25	53.24	37.73
DSA	0.93	4.08	4.35	49.86	44.86

**Table 2:** ICP-OES results of the concentration of metals in the supernatant after desulfurization.

Metal	c/mg L^−1^
As	3.16
Cu	1.14
Cd	0.20
Cr	1.66
Co	0.27
Fe	1500.5
Hg	0.09
Mo	0.41
Ni	1.80
Pb	13.90
Zn	227.82

## Data Availability

Dataset available on request from the authors.
